# Activation of Bicyclic Nitro-drugs by a Novel Nitroreductase (NTR2) in *Leishmania*

**DOI:** 10.1371/journal.ppat.1005971

**Published:** 2016-11-03

**Authors:** Susan Wyllie, Adam J. Roberts, Suzanne Norval, Stephen Patterson, Bernardo J. Foth, Matthew Berriman, Kevin D. Read, Alan H. Fairlamb

**Affiliations:** 1 Division of Biological Chemistry and Drug Discovery, Wellcome Trust Building, School of Life Sciences, University of Dundee, Dundee, Scotland, United Kingdom; 2 Wellcome Trust Sanger Institute, Hinxton, Cambridge, United Kingdom; Washington University School of Medicine, UNITED STATES

## Abstract

Drug discovery pipelines for the “neglected diseases” are now heavily populated with nitroheterocyclic compounds. Recently, the bicyclic nitro-compounds (*R*)-PA-824, DNDI-VL-2098 and delamanid have been identified as potential candidates for the treatment of visceral leishmaniasis. Using a combination of quantitative proteomics and whole genome sequencing of susceptible and drug-resistant parasites we identified a putative NAD(P)H oxidase as the activating nitroreductase (NTR2). Whole genome sequencing revealed that deletion of a single cytosine in the gene for NTR2 that is likely to result in the expression of a non-functional truncated protein. Susceptibility of leishmania was restored by reintroduction of the wild-type gene into the resistant line, which was accompanied by the ability to metabolise these compounds. Overexpression of NTR2 in wild-type parasites rendered cells hyper-sensitive to bicyclic nitro-compounds, but only marginally to the monocyclic nitro-drugs, nifurtimox and fexinidazole sulfone, known to be activated by a mitochondrial oxygen-insensitive nitroreductase (NTR1). Conversely, a double knockout NTR2 null cell line was completely resistant to bicyclic nitro-compounds and only marginally resistant to nifurtimox. Sensitivity was fully restored on expression of NTR2 in the null background. Thus, NTR2 is necessary and sufficient for activation of these bicyclic nitro-drugs. Recombinant NTR2 was capable of reducing bicyclic nitro-compounds in the same rank order as drug sensitivity *in vitro*. These findings may aid the future development of better, novel anti-leishmanial drugs. Moreover, the discovery of anti-leishmanial nitro-drugs with independent modes of activation and independent mechanisms of resistance alleviates many of the concerns over the continued development of these compound series.

## Author Summary

Visceral leishmaniasis (kala-azar) is a serious vector borne disease afflicting people, particularly in parts of Asia, Africa and Latin America. There are approximately 400,000 new cases and an estimated 40,000 deaths each year, making it the second biggest parasitic killer after malaria. We recently discovered that delamanid–an oral nitro-drug used for the treatment of tuberculosis–shows promise for the treatment of leishmaniasis with potential to provide a much needed alternative to the current unsatisfactory anti-leishmanial drugs. Understanding how a drug works is important for selecting the most appropriate partner drugs to be used to increase efficacy and decrease toxicity in patients, to minimise the risk of drug resistance emerging and in designing second generation drugs. Using a combination of biochemical and genetic approaches we have discovered a novel nitroreductase (NTR2) that is necessary and sufficient for the anti-leishmanial activity of delamanid and related experimental drugs containing a nitro-group attached to two fused rings. This enzyme is responsible for activating bicyclic nitro-compounds to form toxic products that kill the parasite. In contrast, the previously identified nitroreductase (NTR1), which specifically activates monocyclic drugs, is not involved in this process. This knowledge can be applied to develop novel treatments for this disease.

## Introduction

New, safer and more effective treatments are required for visceral leishmaniasis (VL), a disease endemic in parts of Asia, Africa and South America. VL results from infection with the protozoan parasites *Leishmania donovani* or *L*. *infantum* and is responsible for ~50,000 deaths per annum, with the number of cases estimated between 200,000 and 400,000 [1]. In 95% of cases, death can be prevented by timely and appropriate drug therapy [2]; however, current treatment options are far from ideal [3]. At present, miltefosine and liposomal amphotericin B are considered the front-line therapies and, while both drugs are considerably more effective than previous treatment options, they have their limitations. The principal drawbacks of amphotericin B include high treatment costs, the requirement of a cold chain for distribution and storage, an intravenous route of administration and unresponsiveness in some Sudanese VL patients [4]. Problems associated with miltefosine, the only oral drug, are its teratogenicity and high potential to develop resistance [5]. Thus, there is a pressing need for better, safer efficacious drugs that are fit-for-purpose in resource-poor settings.

In the search for more effective drugs for VL and other “neglected tropical diseases”, researchers have reassessed the therapeutic value of nitroheterocyclic compounds. Previously avoided in drug discovery programs due to potential mutagenicity and carcinogenicity issues, a nitro-drug is now being successfully used as part of a combination therapy for human African trypanosomiasis (HAT). Nifurtimox-eflornithine combination therapy (NECT) consists of oral treatment with the nitrofuran nifurtimox alongside infusions of eflornithine and has resulted in cure rates of around 97% for the Gambian form of the disease [6]. The 2-substituted 5-nitroimidazole fexinidazole is now in clinical trials for use in the treatment of both HAT and VL [7] (www.dndi.org), and has shown potential for the treatment of Chagas disease [8]. In addition, until recently DND*i* had a nitroimidazole compound (DNDI-VL-2098) [9] and nitroimidazole back-up compounds at an advanced stage of pre-clinical development for use in the treatment of VL (www.dndi.org). Thus, nitroheterocyclic compounds look set to play an important role in the future treatment of these diseases.

Given the new found prominence of nitroheterocyclic drugs, concerted efforts are now being made to elucidate their mechanisms of action. The mode of action of nifurtimox in the trypanosomatids involves reductive activation via a NADH-dependent, type I bacterial-like nitroreductase (NTR, LinJ.05.0660) resulting in the generation of a cytotoxic, unsaturated open-chain nitrile derivative [10]. NTR has also been implicated in the bio-activation of fexinidazole and its sulfonic metabolite, with overexpression of the leishmanial homolog in *L*. *donovani* found to increase sensitivity to fexinidazole sulfone by 15-fold [7]. Indeed, modulation of the NTR levels within the trypanosomatids has been shown to directly affect sensitivity to several nitroheterocyclic compounds *in vitro*, with reduced enzyme activity leading to drug resistance [11–13]. The potential for NTR-related cross-resistance brings into question the rationale of developing further NTR-activated nitro-compounds for the treatment of the trypanosomatid-related diseases. Therefore, it is crucial to determine if any new anti-trypanosomatid nitroaromatics are bio-activated by the NTR at an early stage in development.

Recently, we established that the novel nitroimidazo-oxazine (*R*)-PA-824 and the nitroimidazo-oxazole delamanid (Deltyba, OPC-67683) have potential as effective anti-leishmanial drugs [14,15]. Delamanid is an approved drug for the treatment of multi-drug resistant tuberculosis and (*R*)-PA-824 is the opposite enantiomer of (*S*)-PA-824 (pretomanid) currently in Phase II trials for tuberculosis. The mechanism of action of these bicyclic nitro-compounds does not involve bio-activation via NTR [14,15]. The des-nitro forms of both compounds were inactive against *L*. *donovani*, suggesting that the nitro-group plays a key role in the anti-leishmanial activity of this compound series. This raises the possibility that bio-reduction of (*R*)-PA-824 and delamanid may be mediated by an as yet unidentified nitroreductase within *L*. *donovani*. Here, we describe the identification and characterisation of an FMN dependent NADH oxidoreductase (NTR2) in *L*. *donovani* which is responsible for the bio-activation of (*R*)-PA-824, delamanid and other bicyclic nitro-drugs including DNDI–VL-2098. The broad implications of a novel mechanism for the activation of anti-leishmanial nitroheterocyclic compounds are discussed.

## Results

### (*R*)-PA-824-resistant *Leishmania*

**Fig 1:**
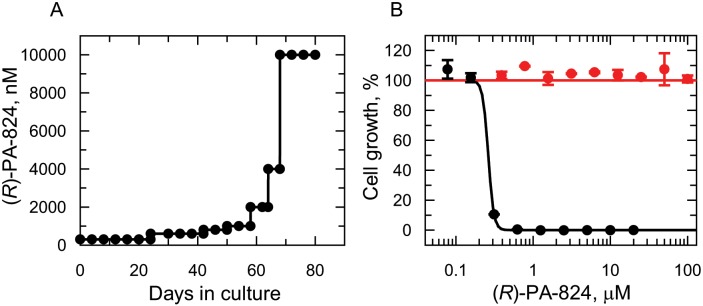
(*R*)-PA-824 resistance *in vitro*. (A) Schematic representation of the generation of a (*R*)-PA-824 resistant cell line in *Leishmania donovani*. Each passage of cells in culture (black circles) is indicated. (B) Dose-response curves for WT (black circles) and RES III resistant cells (red circles). The curves are the non-linear regression fits using a two-parameter EC_50_ equation, yielding EC_50_ values of 262 ± 14 nM and > 100 μM for (*R*)-PA-824 against WT and RES III cells, respectively. Data are the mean ± standard deviation of triplicate cultures in a single experiment.

To investigate the NTR-independent mechanism of action of (*R*)-PA-824, leishmania parasites were selected for resistance against this nitroimidazo-oxazine. Starting with a clonal line of drug-susceptible *L*. *donovani*, promastigotes (the insect stage of the life-cycle) were cultured in the continuous presence of (*R*)-PA-824 for a total of 80 days. Starting at 300 nM (3 x EC_50_, Table 1), three independent cultures were exposed to increasing concentrations of drug until they were routinely growing in 10 μM (*R*)-PA-824 (Fig 1A). Following drug selection, resistant parasites were cloned by limiting dilution. The susceptibility of each cloned cell line to (*R*)-PA-824 was determined and compared to that of wild-type parasites (Fig 1B). All three cloned cell lines were found to be completely refractory to (*R*)-PA-824 at concentrations up to and including 100 μM. Resistance to (*R*)-PA-824 in clone RES III was found to be stable over 45 passages in culture in the absence of drug. In addition, resistance to (*R*)-PA-824 was retained by RES III in the intra-macrophage amastigote stage of the parasite (S1 Fig).

**Table 1:** Sensitivity of WT, (*R*)-PA-824 resistant and transgenic *L*. *donovani* promastigotes to nitroheterocyclic compounds.

	Nitroheterocyclic compounds EC_50_ values, nM (slope factor)^a^							
Cell line	(*R*)-PA-824	(*S*)-PA-824	Delamanid	(*S*)-OPC 67683	DNDI-VL-2098	CGI-17341	Fexinidazole sulfone	Nifurtimox
**WT**	93.6 ± 2.0 (3.5)	2567 ± 50 (3.5)	15.5 ± 0.1 (6.7)	146.9 ± 4.1 (2.9)	30.8 ± 2.8 (1.7)	30.6 ± 1.1 (10.7)	8,200 ± 101 (3.1)	1,800 ± 40 (3.2)
**RES III**	>100,000	>50,000	>50,000	>50,000	>50,000	>20,000	8,900 ± 130 (3.1)	6,020 ± 32 (2.6)
**NTR2** ^**OE**^	8.7 ± 0.3 (2.0)	95 ± 1.6 (2.2)	0.8 ± 0.03 (4.2)	48 ± 3.0 (2.7)	5.4 ± 0.38 (2.4)	1.9 ± 0.1 (8.9)	4600 ± 30 (2.7)	122 ± 8.2 (2.8)
**NTR2** ^**DKO**^	>100,000	>50,000	>50,000	N.D.	>50,000	N.D.	7,880 ± 70 (3.2)	6,400 ± 70 (2.8)

^a^ Data are the weighted means and weighted standard errors of 3 independent experiments. Each individual experiment is derived from a 10 point dilution curve, where each individual sample is measured in triplicate.

N.D–not determined

**Fig 2:**
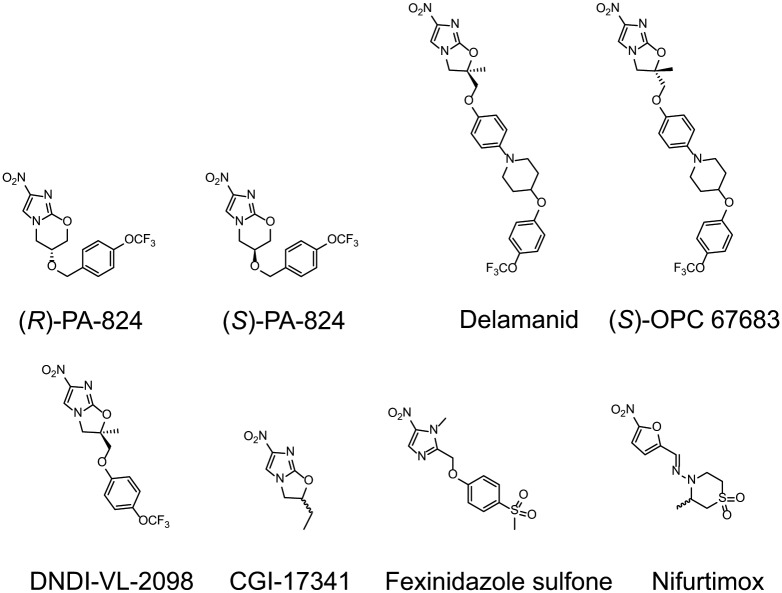
Chemical structures of the bicyclic and monocyclic compounds used in this study. Procedures for the synthesis of DNDI-VL-2098 and CGI-17341 are described in S1 Text.

At this point clone RES III was chosen for in-depth study. Although RES III was completely resistant to (*R*)-PA-824, this clone remained sensitive to drugs from other chemical classes: miltefosine and amphotericin B were equally potent against resistant and parental wild type cells with EC_50_ values of 6.1 ± 0.3 and 5.9 ± 0.5 μM for miltefosine and values of 320 ± 23 and 390 ± 32 nM for amphotericin B against WT and RES III, respectively. However, RES III promastigotes showed marked cross-resistance to a number of structurally-related bicyclic nitroimidazo-oxazine compounds, including delamanid (>3,200-fold) and the preclinical candidate DNDI-VL-2098 (>1,600-fold). In contrast, RES III showed little or no sign of cross-resistance to the monocyclic nitro-drugs, fexinidazole sulfone (1.1-fold) and nifurtimox (3.3-fold) (Table 1). Structures of the compounds tested are shown in Fig 2.

### Proteomic analysis of (*R*)-PA-824 resistant promastigotes

Bio-activation of nifurtimox and fexinidazole sulfone is catalysed by an oxygen-insensitive nitroreductase (NTR) in both *T*. *brucei* [10] and *Leishmania* [7] and loss of, or mutations in, this enzyme has been shown to play a key role in drug resistance mechanisms in the trypanosomatids [16]. Therefore, we hypothesised that should an alternative nitroreductase be involved in the bio-activation of (*R*)-PA-824 in *Leishmania*, changes in this enzyme may be evident in parasites resistant to (*R*)-PA-824. Thus, a comparative proteomic analysis of drug-resistant and WT promastigotes was conducted using stable isotope labelling by amino acids in cell culture (SILAC). WT parasites were grown in modified SDM-79 medium in the presence of normal L-arginine and L-lysine (R0K0) and mixed 1:1 with RES III cells grown for at least 6 cell divisions in the presence of stable isotopes of L-arginine and L-lysine (R6K4) to achieve uniform labelling. Additionally, a label-swap experiment in which the ‘heavy’ and ‘light’ culture media were reversed was also carried out. Cell cultures of WT and RES III were harvested by centrifugation and cell pellets extracted with detergent. Extracted lysates were then pooled, fractionated by SDS-PAGE, digested with trypsin and analyzed by LC-MS/MS. Individual peptides with a heavy to light ratio of 1.0 indicate an equal abundance in both populations.

**Fig 3:**
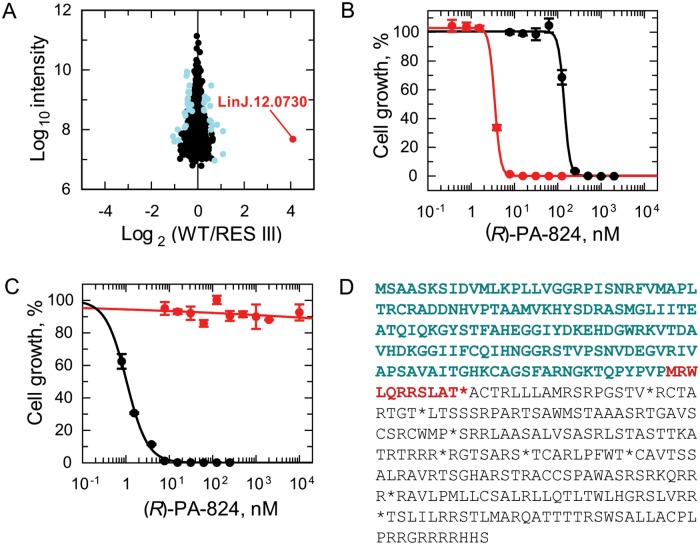
The role of NTR2 in resistance to (*R*)-PA-824 in *Leishmania*. (A) Plot of the proteomics data from (*R*)-PA-824 resistant clone RES III. Each protein identification is represented by a point plotted as the Log_2_ of their heavy-to-light isotope ratio (x axis) versus the Log_10_ value of the intensities of the peptides belonging to each protein (y axis). Proteins plotted in light blue were determined to have significantly different expression levels compared to those in WT parasites, with the most significantly changed protein shown in red (LinJ.12.0730; NADH:flavin oxidoreductase/NADH oxidase, putative). The identity of the proteins found to be consistently over- or under-expressed are listed in S1 Table. (B) Dose-response curve of WT promastigotes (black circles) and promastigotes overexpressing NTR2 (red circles) to (*R*)-PA-824. EC_50_ values of 140 ± 4.6 and 3.5 ± 0.2 nM were determined for WT and NTR2-overexpressing parasites, respectively. Overexpression of NTR2 was confirmed by western blotting (S2 Fig). (C) The susceptibility of RES III parasites (red circles) and RES III parasites overexpressing NTR2 to (*R*)-PA-824 (black circles). RES III parasites were insensitive to (*R*)-PA-824 at concentrations up to and including 100 μM while these promastigotes overexpressing NTR2 returned an EC_50_ value of 1 ± 0.02 nM. Data are the mean ± SD of triplicate cultures. (D) Representation of the frame shift and premature termination of NTR2 translation that results from deletion of a cytosine at genomic position 483544 in (*R*)-PA-824-resistant clones. The frame shift results in a shortened open reading frame and corresponding amino acid sequence (highlighted in bold), with red indicating the frame-shifted part of this sequence.

In the combined proteomic dataset, 2119 proteins were identified by at least one uniquely mapped peptide, prior to filtering the datasets and combining the label-swap experiments. This resulted in the identification of 1472 proteins with a quantifiable expression change between the parental and the RES III cell line. Statistical significance was assessed using significance B, leading to the identification of 38 proteins with significantly altered expression levels compared to the wild type (S1 Table). The most striking change was of a hypothetical NADH:FMN-dependent oxidoreductase (Uniprot: E9AGH7; GeneDB: LinJ.12.0730) identified to be ~16-fold less abundant in RES III parasites (Fig 3A).

### Role of a putative FMN-dependent NADH oxidase in the bio-activation of (*R*)-PA-824

To determine if this putative FMN-dependent NADH oxidoreductase had any role in the bio-activation of (*R*)-PA-824, the open reading frame for LinJ.12.0730 was transfected into WT *L*. *donovani* promastigotes. Overexpression of the enzyme was verified by western blotting (S2 Fig). Parasites overexpressing the enzyme were ~40-fold (EC_50_ = 3.5 nM) more susceptible to (*R*)-PA-824 than WT promastigotes (EC_50_ = 140 nM) (Fig 3B). To further verify the role of this hypothetical oxidoreductase in bio-activation, the enzyme was overexpressed in our drug-resistant cell line RES III (Fig 3C). Overexpressing the oxidoreductase in RES III promastigotes fully restored sensitivity to (*R*)-PA-824 (EC_50_ = 1.0 nM). These data provide compelling evidence that the hypothetical FMN-dependent NADH oxidoreductase, identified in our SILAC studies, is involved in the bio-activation of (*R*)-PA-824 in *L*. *donovani*. Henceforth, this enzyme is known as NTR2 and the previously identified type I oxygen-insensitive nitroreductase as NTR1 (LinJ.05.0660).

### Genomic analysis of *NTR2* in (*R*)-PA-824-resistant parasites

In an attempt to understand the mechanisms involved in the depletion of NTR2 from our drug resistant cell lines and also to identify additional factors that may be involved in resistance, the complete genomes of each independently derived resistant clone (RES I, II and III) were sequenced. Surprisingly, first-pass analysis revealed only 12 single nucleotide polymorphisms (SNPs) resulting in nonsynonymous changes in 3 ORFs in the drug-resistant clones (S2 Table). At this point in our studies, SILAC analysis focused our attention on the role of NTR2 in nitroheterocyclic drug activation and resistance. Complementing this finding, closer examination of *NTR2* and its flanking sequences identified the deletion of a single cytosine (genomic position 483544 on chromosome 12 in LdBPK; C457 in the open reading frame) within *NTR2* that results in a frame shift and premature termination of NTR2 translation (Fig 3D). Despite the fact that each clone appeared to be genetically distinct (S2 Table), this deletion was identified in both allelic copies of *NTR2* in all 3 independently generated resistant clones. The reason for this unusual finding is not clear. Nonetheless, these data, alongside our failure to detect full length NTR2 in RES III parasites (S2 Fig), confirm that each (*R*)-PA-824-resistant clone is effectively NTR2 null and further strengthen our hypothesis that NTR2 is principally responsible for (*R*)-PA-824 bio-activation. A comprehensive analysis of the sequencing data from our (*R*)-PA-824-resistant parasites will be reported in a subsequent publication.

### Can NTR2 activate other nitroheterocyclic drugs?

Having established its role in the bio-activation of (*R*)-PA-284, we assessed the possible role of NTR2 in the activation of other *Leishmania*-active nitroheterocyclic compounds (Table 1). The potencies of these compounds were determined against WT promastigotes and WT transgenic parasites overexpressing NTR2, where hypersensitivity in transgenic parasites is indicative of a compound activated via NTR2. As expected, the (*S*)-enantiomer of PA-824, an anti-tubercular clinical candidate [17], was 27-fold more potent against parasites with elevated levels of NTR2 than WT. Structurally-related compounds including delamanid, CGI-17341 and DNDI-VL-2098 are also activated by NTR2. NTR2-overexpressing parasites showed a <2-fold increase in susceptibility to fexinidazole sulfone suggesting that this nitroimidazole, previously shown to be activated by NTR1, is unlikely to be an efficient substrate for NTR2. However, another known substrate of NTR1, nifurtimox, was 15-fold more potent against NTR2 overexpressing parasites suggesting that this compound may be a substrate of both enzymes in *L*. *donovani*.

### Metabolism of (*R*)-PA-824 and DNDI-VL-2098 in *L*. *donovani*

**Fig 4:**
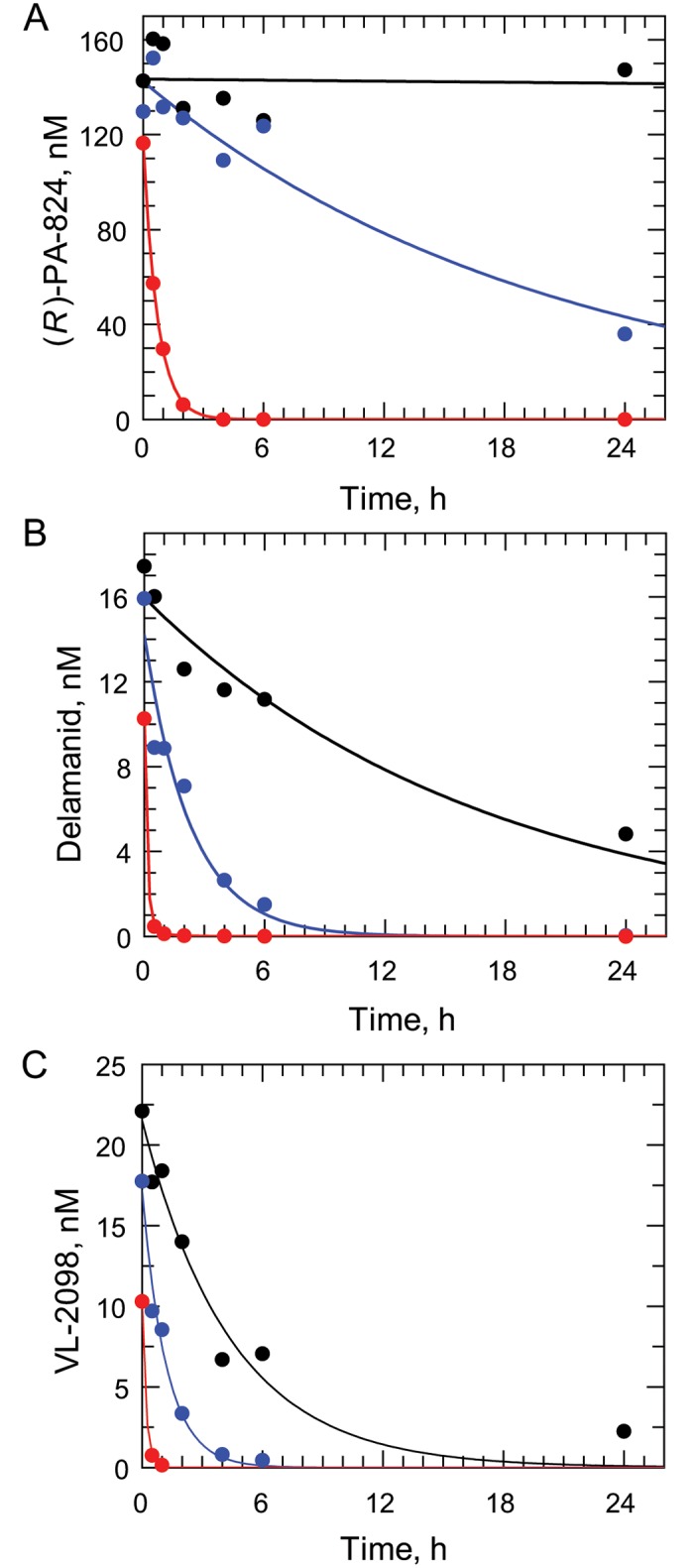
Metabolism of nitroheterocyclics in cultures of WT and NTR2-overexpressing promastigotes. Metabolism of 160 nM (*R*)-PA-824 (A), 15 nM delamanid (B) and 20 nM DNDI-VL-2098 (C) in media alone (black circles), wild type *L*. *donovani* promastigotes (blue circles) and NTR2-overexpressing *L*. *donovani* promastigotes (red circles). The half-life of (*R*)-PA-824 in media alone, in cultures of WT promastigotes and cultures of promastigotes overexpressing NTR2 were >24 h, 14 h and 0.5 h, respectively. DNDI-VL-2098 incubated in media alone had a half-life of 3.1 h and half-lives of 0.83 h and 0.13 h in cultures of WT and NTR2-overexpressing parasites, respectively. The half-life of delamanid was 12 h in media alone and 1.6 h in WT promastigotes. As the disappearance of delamanid in NTR2-overexpressing parasites was plotted to a double exponential decay, two half-lives were calculated as 0.096 h for *k*_1_ and 0.64 h for *k*_2_.

Levels of (*R*)-PA-824 metabolism in WT and NTR2-overexpressing parasites were monitored by UPLC-MS/MS in cultures of promastigotes treated with 160 nM of drug over a 24-h period. (*R*)-PA-824 was essentially stable in culture medium alone with a t_1/2_ of >24 h (Fig 4A). The addition of *L*. *donovani* promastigotes to culture medium resulted in a marked increase in the rate of disappearance of the drug (t_1/2_ = 14 h) associated with the appearance of several drug metabolites. Metabolism was further increased in cultures of parasites overexpressing NTR2 (t_1/2_ = 0.5 h), such that drug levels had dropped below the limit of quantification (0.31 nM) by 4 h. Similar rates of drug metabolism were also observed in cultures incubated with both 15 nM delamanid (EC_50_ value, Fig 4B) and 20 nM DNDI-VL-2098 (10 x EC_50_ value Fig 4C). However, the instability of these compounds in medium alone was higher than those seen with (*R*)-PA-824. This instability can be explained by the fact that delamanid is known to be primarily metabolised in plasma by albumin [18]. Likewise, DNDI-VL-2098 is reportedly unstable in plasma [9], presumably for the same reason.

### Enzymatic analysis of recombinant NTR2

**Fig 5:**
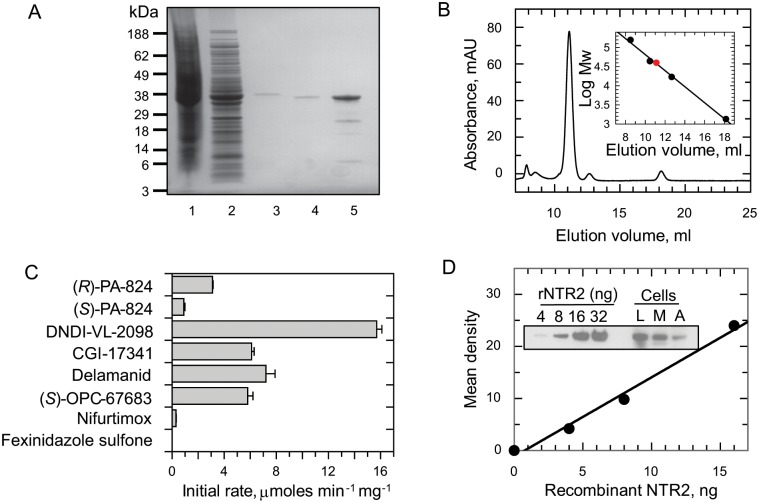
Characterisation of LdNTR2. (A) Purification of recombinant LdNTR2 from *E*. *coli* BL21(DE3)pLysS [pET15b-LdNTR2]. Lane 1, insoluble fraction; lane 2, soluble fraction; lane 3, pooled fractions from Ni^2+^-affinity chromatography; lane 4, soluble protein following removal of histidine tag; and lane 5, pooled fractions from size-exclusion chromatography. MALDI analysis confirmed that the minor bands represent NTR2 degradation products. (B) Gel filtration profile of the LdNTR2. The inset shows a plot of elution volume against the log molecular mass (MW) of a standard protein mixture (black circles). The red circle represents the elution volume of NTR2. (C) Metabolism of nitroheterocyclic compounds by recombinant NTR2. Initial rates of metabolism were measured in assays containing 100 μM nitro-compound, 100 μM NADPH and 500 nM NTR2. Rates of metabolism with NADPH and NADH alone were 0.0124 ± 0.001 and 0.0084 ± 0.0006 μmol min^-1^ mg^-1^, respectively. Rates represent the mean ± SD of triplicate measurements. (D) Immunoblots of whole cell extracts (equivalent of 5×10^6^ parasites in each lane) from *L*. *donovani* mid-log promastigotes (L), metacyclic promastigotes (M) and axenic amastigotes (A) were probed with LdNTR2-specific polyclonal antiserum. Known amounts of purified recombinant LdNTR2 were loaded as standards for the quantification of the cellular levels of NTR2.

To further study the substrate specificity of NTR2, the recombinant enzyme was expressed and purified to homogeneity in three chromatographic steps to obtain a yield of 15 mg l^−1^ of a yellow product (Fig 5A), indicative of a flavoprotein. Using an established spectrophotometric method, FMN was confirmed as the bound co-factor in NTR2 [19]. Analysis of the recombinant protein by size-exclusion chromatography revealed that NTR2 elutes primarily as a monomer at ∼40 kDa (Fig 5B), close to the predicted molecular mass of 39.6 kDa (Fig 5B). This was confirmed by MS to be 39.4 kDa for the recombinant protein by MALDI–TOF analysis.

Our preliminary studies indicate that this enzyme was able to utilise NADH or NADPH as a reductant. The ability of NTR2 to reduce a variety of nitroheterocyclic compounds was then assessed in the presence of 100 μM NADPH (Fig 5C). The highest rates of activity were observed with the bicyclic nitro-compounds structurally related to (*R*)-PA-824. Further, the highest rates of NTR2 metabolism broadly correlate with the most potent anti-leishmanial compounds, DNDI-VL-2098 (15.7 ± 0.4 μmol min^-1^ mg^-1^) and delamanid (7.2 ± 0.7 μmol min^-1^ mg^-1^). In keeping with our overexpression studies in Table 1, recombinant NTR2 was able to reduce nifurtimox, albeit at a comparatively low rate (0.30 ± 0.02 μmol min^-1^ mg^-1^) but showed little activity with fexinidazole sulfone (0.004 ± 0.0002 μmol min^-1^ mg^-1^).

### Quantitation of cellular NTR2 levels

Using a NTR2-specific polyclonal antiserum generated against our purified recombinant protein, we were able to confirm that NTR2 is expressed in all developmental stages of the *Leishmania* parasite by probing an immunoblot of whole cell lysates (Fig 5D). Single bands of approximately 40 kDa, close to the predicted molecular mass of NTR2 (39.6 kDa), were detected in cell lysates of log phase promastigotes (the dividing insect stage), metacyclic promastigotes (the insect stage infective to mammals) and axenic amastigotes (the intracellular mammalian stage). The cellular concentration of NTR2 in each of these parasite stages was determined by densitometry. NTR2 levels in each developmental stage were found to be remarkably similar with concentrations of 1.70 μM, 1.73 μM and 1.75 μM in promastigotes, metacyclics and amastigotes, respectively.

### Cellular localisation of NTR2

**Fig 6:**
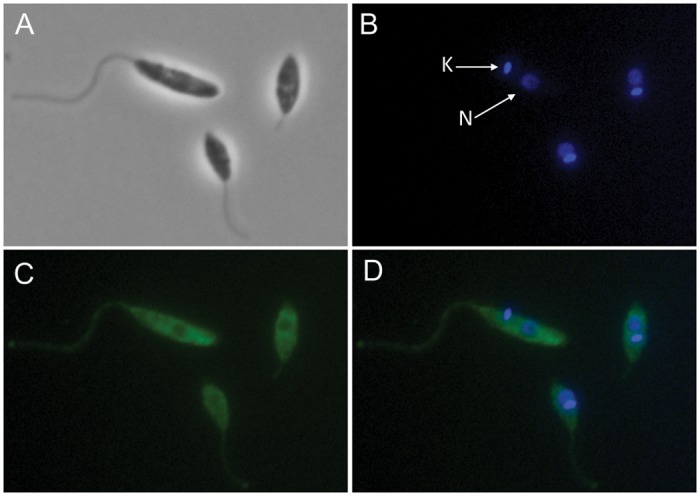
Cellular localisation of NTR2. Phase contrast images of *L*. *donovani* promastigotes (A), promastigotes stained with DAPI (4,6-diamidino-2-phenylindole) (B), immunofluorescence staining of promastigotes with NTR2 anti-serum (FITC) (C) and a merged FITC and DAPI image (D). The kinetoplastid (K) and nucleus (N) are indicated in the DAPI stained image.

Immunofluorescence studies confirm that *L*. *donovani* NTR2 localises to the cytosol of mid-log promastigotes (Fig 6). Staining of promastigotes with an anti-NTR2 polyclonal antibody showed extensive and even staining throughout the cells, except for the nucleus and kinetoplast, demonstrating the cytosolic location of this enzyme (Fig 6C and 6D).

### Assessing the essentiality of NTR2

**Fig 7:**
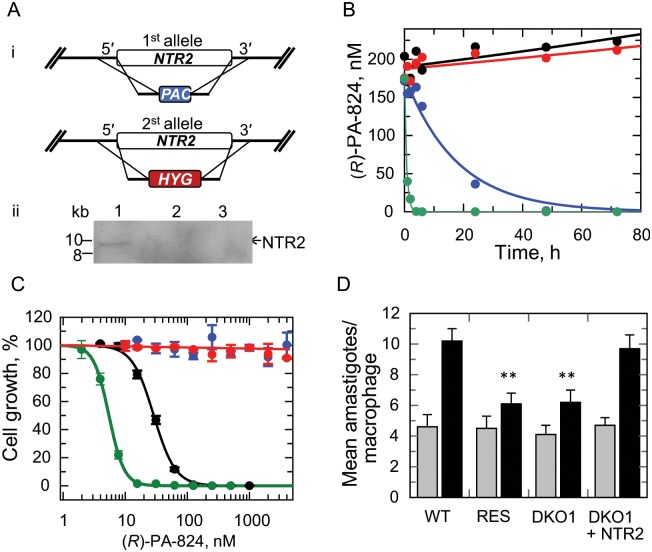
Modulation of NTR2 levels and its effect on (*R*)-PA-824 potency, compound metabolism and infectivity. (A) (i) Schematic representation of the stepwise generation of the *NTR2* DKO cell line in *L*. *donovani*. One allele of *NTR2* was replaced with the puromycin resistance gene (PAC) by homologous recombination; the remaining allele was replaced with a hygromycin resistance gene (HYG) by homologous recombination resulting in a NTR2 null cell line. (ii) Southern-blot analysis of XhoI-digested genomic DNA (∼5 μg) from wild-type *L*. *donovani* (LdBOB) cells (lane 1), NTR2-double knockout clone 1 (DKO1) cells (lane 2) and NTR2-double knockout clone 2 (DKO2) cells (lane 3). The *NTR2* ORF probe shows allelic *LdNTR2* at 10 kb. (B) EC_50_ values were determined for (*R*)-PA-824 against WT (black), DKO1 (red), DKO2 parasites (blue) and DKO1 parasites with an NTR2 add-back (green). An EC_50_ value of 115 ± 3 nM was determined for (*R*)-PA-824 against WT parasites, DKO1 and DKO2 were unaffected by the drug at concentrations up to and including 10 μM, while an EC_50_ value of 5.5 ± 0.03 nM was determined for DKO1 parasites expressing an NTR2 add-back. (C) Metabolism of 160 nM (*R*)-PA-824 in media alone (black circles), wild type *L*. *donovani* promastigotes (blue circles), DKO parasites (red circles) and DKO parasites plus NTR2 add-back (green circles). The half-life of (*R*)-PA-824 metabolism in cultures of WT parasites is 12.5 h, while the half-life of DKO parasites with an NTR2 add-back is 0.5 h. (D) Mean numbers of WT, DKO (clone 1) and RES (clone RES III) amastigotes infecting mouse peritoneal macrophages. Grey bars, invasion after 24 h; black bars, replication after 72h. Differences in the replication of WT versus RES and DKO amastigotes in macrophages were confirmed as highly significant (P values equal to 0.0057 and 0.0087, respectively) using an un-paired student t-test (**).

The studies described above show that loss of NTR2 is strongly associated with resistance to (*R*)-PA-824. However, this does not exclude the possibility that additional genes may also be involved. To address this issue, we investigated the impact of NTR2 loss by gene deletion in a WT genetic background. Thus, *NTR2* null parasites were generated by classical gene replacement. Both copies of the *NTR2* gene were sequentially replaced with hygromycin and puromycin drug resistance genes. Southern blot analysis of genomic DNA from putative double knockout (DKO) cells confirmed that they were *NTR2* null (Fig 7A). Loss of both copies of *NTR2* had no obvious effects on the viability of these parasites with DKO promastigotes growing at the same rate in culture as WT and achieving similar cell densities. DKO parasites were found to be completely refractory to (*R*)-PA-824 at concentrations up to and including 100 μM (Fig 7C), as found in our resistant lines obtained by drug selection. Similar results were found for (*S*)-PA-824, delamanid and DNDI-VL-2098 (Table 1). Susceptibility to fexinidazole sulfone remained unchanged and susceptibility to nifurtimox decreased marginally in good agreement with RES III. Adding back an exogenous copy of NTR2 to DKO null parasites entirely recovered sensitivity to (*R*)-PA-824 demonstrating that NTR2 is necessary and sufficient for activation of toxicity with compounds such as (*R*)-PA-824, delamanid or DNDI-VL-2098.

The impact of NTR2 deletion on drug metabolism was determined by measuring the concentration of (*R*)-PA-824 in cultures of WT and DKO over a 24-h period. Samples of culture were removed at defined intervals and the supernatants analysed by UPLC-MS/MS, as previously described (Fig 7B). WT parasites metabolised (*R*)-PA-824 at a similar rate to that seen in our earlier study (t_1/2_ = 12.5 h). In contrast, rates of metabolism in medium alone and in cultures of DKO promastigotes were negligible over the same 24-h period. The addition of an NTR2 add-back to DKO parasites recovered the ability of these cells to metabolise (*R*)-PA-824 (t_1/2_ = 0.5 h). These data confirm that NTR2 alone is necessary and sufficient for metabolic conversion of bicyclic nitro-drugs.

Loss of functional *NTR2* did not have a material effect on the ability of DKO or RES metacyclic promastigotes to infect peritoneal macrophages, as determined by comparing the mean numbers of amastigotes per infected macrophage to that seen in WT-infected macrophage cultures 24h following infection (Fig 7D). However, there did appear to be a moderate but statistically significant effect on the ability of NTR2-deficient parasites to replicate within peritoneal macrophages with mean numbers of amastigotes per infected macrophage considerably lower in DKO and RES cultures at 72h. The reduced ability of NTR2 null amastigotes to replicate within macrophages was entirely alleviated by the addition of an NTR2-addback. Collectively, these data suggest that, while NTR2 is not essential for *L*. *donovani* survival, null parasites do appear to suffer a moderate but statistically significant loss of “fitness” in macrophage infections that may have implications for the propagation of NTR2-related drug resistance in the field.

## Discussion

Drug discovery pipelines for the “neglected diseases” are now heavily populated with nitroheterocyclic compounds. Following the success of nifurtimox as part of NECT [6] and the re-discovery of fexinidazole [7,8,20], researchers have been quick to recognise and exploit the therapeutic potential of these compound classes. However, the development of multiple compounds with a likely shared mode of action for any disease indication is not without significant risk. An over-emphasis on one compound class can leave drug pipelines vulnerable to multiple compound failures associated with a single resistance mechanism. Specifically, it is well established that parasites resistant to one nitro-drug are often cross-resistant to a second [13]; for example nifurtimox-resistant *T*. *brucei* are cross-resistant to fexinidazole and vice versa [11,16]. Here, cross-resistance has been largely explained by a common, nitroreductase-related mechanism of drug activation [13,21]. This has made researchers wary of developing further nitro-compounds for the treatment of the trypanosomatid diseases. In this study we have confirmed that several bicyclic nitro-drugs, either in preclinical development or demonstrating promising anti-leishmanial activity, are not activated via NTR1, known to activate monocyclic nitro-compounds nifurtimox, benznidazole and fexinidazole [7,10,12,22]. Importantly, resistance to bicyclic nitro-compounds in *Leishmania* promastigotes does not result in striking levels of cross-resistance to NTR1-activated compounds. The discovery of anti-leishmanial nitro-drugs with independent modes of activation and independent mechanisms of resistance alleviates many of the concerns over the continued development of these compound series.

It is worth emphasizing the power of using orthogonal approaches such as pharmacology combined with genomics and proteomics to elucidate mechanisms of drug action. Several lines of evidence presented here establish that the primary enzyme target for metabolic activation of bicyclic nitro-compounds in *Leishmania* is NTR2, an NAD(P)H-dependent flavoprotein. First, whole genome sequencing and SILAC proteomic analysis confirmed that *Leishmania* promastigotes, resistant to (*R*)-PA-824 and cross-resistant to a number of bicyclic nitro-drugs, are effectively NTR2 null. Second, re-introduction of NTR2 into (*R*)-PA-824-resistant parasites restored drug susceptibility while overexpression of NTR2 resulted in hypersensitivity to bicyclic nitro-drugs. Third, and perhaps the most compelling evidence that NTR2 is primarily responsible for metabolic activation of these compounds, is the complete abrogation of susceptibility in NTR2 null parasites.

Increased metabolism of (*R*)-PA-824, delamanid and DNDI-VL-2098 in promastigotes overexpressing NTR2 and the absence of metabolism in NTR2 DKO cultures suggests that NTR2 catalyses metabolic conversion of these compounds. In *Mycobacterium tuberculosis* metabolism of (*S*)-PA-824 is catalyzed by an unusual deazaflavin-dependent nitroreductase (Ddn) [23–25], an enzyme which is absent in *Leishmania spp*. Incubation of (*S*)-PA-824 with recombinant *M*. *tuberculosis* Ddn leads to the formation of three primary metabolites, the most abundant being (*S*)-des-nitro-PA-824 [25,26]. Des-nitro-formation in this bacterium leads to the concomitant release of reactive nitrogen species, including nitric oxide. Transcriptional profiling suggests that respiratory poisoning by nitric oxide is likely to be central to the anti-mycobacterial action of (*S*)-PA-824 under hypoxic conditions [27]. Further studies will be required to elucidate the chemical identity of the metabolite(s) resulting from NTR2 bio-activation of bicyclic nitro-drugs and their role(s) in parasite killing.

The endogenous function of NTR2 in *Leishmania* remains to be determined. BLAST searches of NTR2 revealed high similarity to prokaryotic alkene reductases for the “old yellow enzyme” family. Members of this “ene”-reductases family catalyze a diverse range of reactions, usually on substrates with an α/β unsaturated carbonyl group [28–30]. The closest orthologue of NTR2 in *Trypanosoma cruzi* is the enzyme prostaglandin F2α synthase, also known as old yellow enzyme, which shares 44% identity with *L*. *donovani* NTR2. This NAD(P)H-dependent oxidoreductase has been implicated in both the mechanisms of action of, and resistance to, benznidazole and nifurtimox in the American trypanosome [31]. There is no enzyme equivalent to NTR2 in the genome of *T*. *brucei*, perhaps explaining the conspicuous lack of potency of bicyclic nitro-drugs against these parasites [15].

Our future studies will involve a comprehensive kinetic and structural characterisation of NTR2 and an elucidation of the active drug metabolites responsible for cell death. Understanding the binding mode of compounds in the active site of NTR2 may facilitate the design of improved bicyclic nitro-drugs for the treatment of VL.

## Materials and Methods

### Ethics statement

All animal experiments were approved by the Ethical Review Committee at the University of Dundee and performed under the Animals (Scientific Procedures) Act 1986 (UK Home Office Project Licence PPL 70/8274) in accordance with the European Communities Council Directive (86/609/EEC).

### Cell lines and culture conditions

The clonal *Leishmania donovani* cell line LdBOB (derived from MHOM/SD/62/1S-CL2D, originally isolated from a patient in the Sudan in 1962) [32] was grown as promastigotes at 26°C in modified M199 media supplemented with FCS certified free from mycoplasma (6).

### Test compounds

(*R*)-PA-824 and (*S*)-PA-824 were synthesized in-house following published procedures [7,15]. Fexinidazole sulfone was prepared either by oxidation of fexinidazole [33], or by modification of a published method [34]. DNDI-VL-2098 was prepared by adapting the reported syntheses of related compounds [35]. Methods for the synthesis of delamanid and (*S*)-OPC-67683 are described in detail in our recent publication [14]. CGI-17341 was prepared using a modification of a previous method [36]. The purity of all synthesized compounds was determined by liquid chromatography-mass spectrometry and found to be >95%. Where appropriate, the optical rotation of chiral compounds was checked against literature values and in all cases was found to be in good agreement. Full experimental details and analytical data for the synthesis of DNDI-VL-2098 and CGI-17341 are reported in S1 Text.

### *In vitro* drug sensitivity assays

Drug sensitivity assays were carried out in triplicate promastigote cultures exactly as previously described [15]. Data were fitted by non-linear regression to a two-parameter EC_50_ equation using GraFit version 5.0.13.

### Generation of drug-resistant parasites

(*R*)-PA-824 resistant lines were generated by sub-culturing a freshly cloned line of wild-type *L*. *donovani* in the continuous presence of this compound. Starting at a sub-lethal concentration of 300 nM (*R*)-PA-824, the drug concentrations in 3 independent cultures were increased in a step-wise manner, usually by 2-fold. After a total of 80 days in culture, when promastigotes were able to survive and grow in 10 μM (*R*)-PA-824, the resulting cell lines were cloned by limiting dilution in the absence of (*R*)-PA-824. One clone (RES III) was selected for further biological studies.

### SILAC labelling, cell culture and sample preparation

SILAC SDM-79 medium (SDM-79^SILAC^) was prepared as previously described [37]. Briefly, SDM-79^SILAC^ was supplemented with either normal isotopic abundance L-Arginine and L-Lysine (SDM-79^SILAC-L^), or with L-arginine.HCl [U-^13^C_6_] and L-lysine.2HCl [4,4,5,5-D4] (SDM-79^SILAC-H^, CK Gas Products, UK) at the same concentration as described in the original SDM-79 formulation [38].

For SILAC labelling of cultures, LdBOB WT or (*R*)-PA-824 resistant promastigotes, in the log phase of growth, were washed 3 times with phosphate-buffered saline, and resuspended at 1 × 10^4^ cells ml^-1^ in either SDM-79^SILAC-L^ or SDM-79^SILAC-H^. Cells were passaged every 2 days and grown for a total of 10 days under labelling conditions. Cells (5×10^7^) were harvested by centrifugation (10 min, 4°C, 1600 *g*) and washed twice in PBS prior to being resuspended in Laemmli buffer (Bio-Rad Laboratories) and heated at 95°C for 10 min. The equivalent of 5×10^6^ cells of each cell sample (WT and (*R*)-PA-824 resistant promastigotes) were pooled together and then subjected to electrophoresis on a 4–12% NuPAGE SDS/PAGE gel. When the sample had entered approximately 2 cm into the gel, electrophoresis was stopped and the gel stained with Instant Blue (expedeon). Sample lanes were excised and subjected to in-gel digestion for 18 h at 37°C with 12.5 μg ml^-1^ Trypsin Gold (Promega) in 10 mM NH_4_HCO_3_ and 10% acetonitrile. Tryptic peptides were recovered in 45% acetonitrile, 1% formic acid and lyophilized prior to analysis.

### Mass spectrometry data acquisition and processing—SILAC

Liquid chromatography tandem mass spectrometry was performed by the Proteomic Facility at the University of Dundee. Tryptic peptides were separated on a fully automated Ultimate U3000 Nano RSL Cnano system (Thermo Scientific) fitted with a 0.1 × 2 cm PepMap C18 trap column and a 75 μm × 50 cm reverse phase PepMap C18 nanocolumn (Thermo Scientific). Samples were loaded in 0.1% formic acid (buffer A) and separated using a binary gradient consisting of buffer A and buffer B (80% acetonitrile, 0.08% formic acid). Peptides were eluted with a linear gradient from 2 to 40% buffer B over 124 min. The HPLC system was coupled to an LTQ Orbitrap Velos Pro mass spectrometer (Thermo Scientific) equipped with a Proxeon nanospray ion source. The mass spectrometer was operated in data dependent mode to perform a survey scan over a range 335–1800 m/z in the Orbitrap analyzer (R = 60,000), with each MS scan triggering ten MS2 acquisitions of the ten most intense ions. The Orbitrap mass analyzer was internally calibrated on the fly using the lock mass of polydimethylcyclosiloxane at m/z 445.120025.

Data was processed using MaxQuant version 1.5.0 which incorporates the Andromeda search engine [39,40]. Proteins were identified by searching a protein sequence database containing *L*. *infantum* annotated proteins (downloaded from UniProt, http://www.uniprot.org/proteomes/UP000008153) supplemented with frequently observed contaminants (porcine trypsin, bovine serum albumin and human keratins). Initial MS tolerance was set as 4.5 ppm with the MS/MS tolerance set at 0.5 Da. Cysteine carbamidomethylation was set as a fixed modification with protein *N*-acetylation and methionine oxidation as variable modifications. Peptides were required to be a minimum of 7 amino acids in length with only the uniquely mapped peptides used in the calculation of SILAC ratios. The minimum H/L ratio count was set to be 1 and peptide and protein false discovery rates of 0.01 were calculated by searching a database of reversed sequences. The SILAC ratios of proteins identified in both label swap experiments were averaged, whereas reported H/L ratios identified only in one experiment were included if the H/L percentage variability was <100% [41]. Differential expression was assessed using a significance B test built into Perseus v.1.5.0 with a Benjamini–Hochberg FDR threshold of 0.01 [40].

### Generation of overexpression constructs

The primers used to generate constructs for genetic manipulation and protein expression (S3 Table) were designed using the *L*. *infantum* genome sequence (tritrypdb.org). Primers were designed against a putative NADH:flavin oxidoreductase/NADH oxidase (LinJ.12.0730). The accuracy of all assembled constructs was verified by sequencing.

Ld*NTR2* overexpression vectors were generated by amplifying the gene from genomic DNA using the Ld*NTR2*-BamHI sense and antisense primers for cloning into pIR1-SAT and Ld*NTR2*-SmaI sense and Ld*NTR2*-XbaI antisense for cloning into pX63-3HA. PCR products were then cloned into the pCR-Blunt II-TOPO vector (Invitrogen) and sequenced. The pCR-Blunt II-TOPO-gene constructs were then digested with appropriate restrictions enzymes and the fragments cloned into either the pIR1SAT or pX63-3HA expression vectors.

### Assembly of knockout constructs

*NTR2* gene replacement cassettes were generated by amplifying a region of DNA encompassing the 5´-untranslated region (UTR), open reading frame (ORF) and 3´-UTR of LdBOB *NTR2* from genomic DNA with primers 5´UTR-NotI_s and 3´UTR-NotI_as, using *Pfu* polymerase. This sequence was then used as a template for the amplification of the individual regions used in the assembly of replacement cassettes containing the selectable drug resistance genes puromycin *N*-acetyl transferase (*PAC*) and hygromycin phosphotransferase (*HYG)*, exactly as previously described [42].

### Generation of LdBOB transgenic cell lines

Mid-log-phase *L*. *donovani* promastigotes (LdBOB) were transfected with overexpression constructs using the Human T-Cell Nucleofector kit and the Amaxa Nucleofector electroporator (program V-033). Following transfection, cells were allowed to grow for 16–24 h in modified M199 medium [32] with 10% fetal calf serum prior to appropriate drug selection (100 μg nourseothricin μg ml^−1^, hygromycin 50 μg ml^-1^, puromycin 20 μg ml^-1^ and 100 μg G418 ml^−1^). Cloned cell lines were generated by limiting dilution, maintained in selective medium, and removed from drug selection for one passage prior to experiments.

### Infectivity assays

In-macrophage infectivity assays were carried out using starch-elicited mouse peritoneal macrophages harvested from BALB/c mice [7] and metacyclic promastigotes, as previously described [12].

### Metabolism of (*R*)-PA-824, delamanid and DNDI-VL-2098 in *L*. *donovani* promastigotes

Metabolism studies were performed at 160 nM (*R*)-PA-824, 15 nM delamanid and 20 nM DNDI-VL-2098 in culture medium alone and in the presence of either wild type, NTR2 null or NTR2 overexpressing *L*. *donovani* promastigotes (1 x 10^7^ parasites ml^-1^). At 0, 0.5, 1, 2, 4, 6, 8 and 24 h aliquots were removed, precipitated by addition of a 2-fold volume of acetonitrile and centrifuged (1,665 x g, 10 min, RT). The supernatant was diluted with water to maintain a final solvent concentration of 50% and stored at -20°C prior to UPLC-MS/MS analysis, as described below. Data were processed using GraFit (version 7.0.2; Erithacus software) and fitted to a single exponential decay (with the exception of delamanid incubated with NTR2^OE^ parasites which were fitted to a double exponential decay) and the half life (t_1/2_) was calculated from the elimination rate constant (*k*):

$$t_{1/2}=\frac{ln2}{k}$$

### Metabolite identification—UPLC-MS/MS methods

UPLC-MS/MS was performed on a Waters Acquity UPLC interfaced to a Waters Xevo TQ-S MS (Waters, Manchester, UK). Chromatographic resolution was achieved on a 2.1 x 50 mm Acquity BEH C18, 1.7 μm column which was maintained at 40°C with an injection volume of 8 μl. The mobile phase consisted of A: deionized water plus 0.01% (v/v) formic acid and B: acetonitrile plus 0.01% (v/v) formic acid at a flow rate of 0.6 ml min^-1^. The initial gradient was 5% B held for 0.5 min before increasing to 95% B from 0.5–2 min, where it was held from 2–2.6 min before decreasing back to 5% B from 2.6–3 min. Mass spectra were obtained using electrospray ionization (ESI), in positive ion mode with the following conditions: capillary 3.5 kV; desolvation temperature 600°C; source temperature 150°C; desolvation gas flow (nitrogen) 1000 l h^-1^ and collision gas (argon) gas of 0.15 ml min^-1^. Multiple reaction monitoring (MRM) was performed using the transition 359.87 > 174.96, cone voltage of 5 V and collision energy of 26 V for (*R*)-PA-824, the transition 535.02 > 351.08, cone voltage of 16 V and collision energy of 33 V for delamanid and 359.92 > 230.92 at a cone voltage of 5 V and collision energy of 12 V for VL-2098 Data was processed using the TargetLynx feature of Mass Lynx v4.1.

### Cloning, expression and purification of recombinant NTR2

Ld*NTR2* was amplified from genomic DNA using the primers Ld*NTR2*-NdeI_s and Ld*NTR2*-BamHI_s (S3 Table). The resulting PCR product was then cloned into the pCR-Blunt II-TOPO vector (Invitrogen) and sequenced. The pCR-Blunt II-TOPO-gene constructs were then digested with appropriate restriction enzymes and the fragment cloned into the multiple cloning site of the pET-15b-TEV expression vector. The resulting pET15b-NTR2 expression construct was transformed into BL21 (DE3)pLysS competent cells and recombinant expression was carried. Overnight starter cultures were used to inoculate one litre of LB media supplemented with 50 μg ml^-1^ ampicillin. These cultures were grown at 37°C with shaking (200 rpm) until an OD_600_ of 0.7 was reached. At this point the temperature was dropped to 22°C, expression induced by the addition of 0.5 mM IPTG and cultures incubated for a further 12 h. Cells were harvested by centrifugation (5090 *g*, 30 min at 4°C) and resuspended in buffer A (50 mM Na^+^ phosphate, 150 mM NaCl, 25 mM imidazole pH adjusted to 7.5) supplemented with an EDTA free protease inhibitor cocktail tablet (Roche), DNase I and lysozyme. Cells were disrupted using a Constant Systems, continuous flow cell disruptor at 30 kpsi and soluble protein recovered after centrifugation (40,000 × g, 30 min at 4°C). Soluble protein was loaded onto a 5-ml HisTrap column and eluted with an increasing gradient of imidazole (25–500 mM) in buffer A. Fractions containing the protein of interest were pooled prior to the addition of TEV protease and dialysed into 50 mM Na^+^ phosphate buffer containing 150 mM NaCl overnight at 4°C using a 10 kDa MWCO dialysis cassette. The protein was loaded onto the 5-ml HisTrap column and then eluted with buffer A. Fractions containing the protein were loaded onto a Superdex 75 26/60 column equilibrated in buffer B (50 mM tris containing 150 mM NaCl, pH 7.5) and separated. After analysis by SDS-PAGE, fractions containing NTR2 were pooled and concentrated for storage in buffer B containing 10% glycerol and supplemented with 1 mM DTT. Analytical size exclusion was carried out in buffer B on a Superdex 75, 10/300 column injecting 100 μl of 2 mg ml^-1^ protein and comparing the elution volume to that of the BioRad gel filtration standards. Final purity was assessed by SDS-PAGE, MALDI-TOF and tryptic mass fingerprinting.

### NTR2 enzymatic activity

NTR2 activity was measured by following the change in absorbance at 340 nm due to NADPH oxidation. A reaction mixture (1 ml) containing 50 mM HEPES, pH 7.0 and 100 μM NADPH was incubated at 25°C for 1 min. NTR2 was added to a final concentration of 500 nM and the background rate of NADPH oxidation was measured for 1 min. The reaction was initiated by the addition of 100 μM of nitroheterocyclic test compounds and initial rates of NADPH oxidation in the presence of these compounds were measured. Enzyme activity was calculated using ε = 6220 M^−1^ cm^−1^ and reported in μmol min^-1^ mg^-1^.

### Quantitation of cellular levels of NTR2 in cell lysates

Mid-log promastigotes, metacyclic promastigotes and axenic amastigotes were harvested by centrifugation (10 min, 4°C, 1600*g*) and washed twice in PBS. Cells (5×10^7^) were resuspended in 100 μl Laemmli buffer (Bio-Rad Laboratories) and heated at 95°C for 10 min. The equivalent of 5×10^6^ cells were separated by SDS-PAGE on a 4–12% NuPAGE gel, alongside known amounts of purified recombinant NTR2 (4–32 ng). Cellular proteins were transferred on to Protran nitrocellulose membrane (Whatman) by electrotransfer. Membranes were probed with primary rabbit antisera generated against purified, recombinant NTR2 (1:3000 dilution, Davids Biotechnologie, Regensburg, Germany) prior to probing with an HRP (horseradish peroxidase)-conjugated goat anti-rabbit polyclonal secondary serum (1:5000; BioRad). The blot was developed using ECL detection reagent kit (GE Healthcare) and exposed to Amersham Hyperfilm ECL (GE Healthcare). The developed film was scanned and the protein bands were quantified by densitometry with ImageJ (NIH). Cell volumes, used to determine the intracellular concentration of NTR2, were measured using a Schärfe Systems CASY cell counter yielding 2.86 ± 0.02, 2.5 ± 0.01 and 1.74 ± 0.04 μl (10^8^ cells)^-1^ for LdBOB mid-log promastigotes, metacyclic promastigotes and axenic amastigotes, respectively. Total protein in 2% SDS was determined using the Pierce BCA Protein Assay Kit and found to be similar across all developmental stages (0.82, 0.78 and 0.68 mg (10^8^ cells)^-1^).for mid-log promastigotes, metacyclic promastigotes and axenic amastigotes, respectively).

### Immunofluorescence studies

Mid-log *L*. *donovani* promastigotes were washed twice in PBS before being fixed in 2% (w/v) paraformaldehyde in PBS (0.15 M NaCl, 5 mM potassium-phosphate buffer, pH 7.4). Fixed parasites were then treated with Triton X-100 (0.1%) for 10 min, prior to the addition of 0.1M glycine for an additional 10 min. Parasites were then washed with PBS and air-dried onto polylysine coated microscope slides. Slides were then blocked by incubation in 50% (v/v) foetal calf serum (FCS), PBS for 10 min prior to incubation in *L*. *donovani* NTR2 antiserum diluted 1:50 in PBS containing 5% FCS for 1 h at room temperature. Following washing in PBS, slides were incubated for a further 1 h in fluorescein isothiocyanate-conjugated goat anti-rabbit secondary antibody diluted 1:200 in PBS. Slides were washed again in PBS before being mounted using the SlowFade Light Antifade Kit with 4,6-diamidino-2-phenylindole (DAPI; Molecular Probes), as instructed by the manufacturers.

### Whole genome sequencing and genomic variant analysis

Genomic DNA was prepared from *L*. *donovani* (*R*)-PA-824 resistant clones RES I, II and III and the wild-type parental strain. For each sample, 1.5–2 μg of genomic DNA was used to produce amplification-free Illumina libraries of 400–600 base pairs (bp) length [43]. Sequencing was carried out on an Illumina HiSeq 2000 sequencer according to the manufacturer’s standard sequencing protocol and yielded 38.0–48.9 million reads of 100 bp length per library. The resulting data sets represented a nominal sequencing coverage of the *L*. *donovani* genome (32.4 Mb) of 117.4- to 150.9-fold. The Illumina data were aligned against the *L*. *donovani* BPK282A1 reference genome [44] assembly using SMALT v0.7.4 (http://www.sanger.ac.uk/resources/software/smalt/) employing an exhaustive search (-x) and with parameters wordlen = 13 (-k), skipstep = 2 (-s), minscor = 0.60 (-m), and insertmax = 1000 (-i). Variants were called using SAMtools v0.1.19 mpileup (-Q 15 for baseQ/BAQ filtering) and BCFtools [45]. The raw sequence data are available under the study number PRJEB5833 and the following accession numbers at the European Nucleotide Archive (http://www.ebi.ac.uk/ena): LdWT: ERS421640; Ld PA824R-I: ERS421641; Ld PA824R-II: ERS421642; Ld PA824R-III: ERS421643.

## Supporting Information

S1 Text Procedures for the synthesis of DNDI-VL-2098, CGI-17341 and delamanid. (DOCX)

S1 Fig Susceptibility of RES III and WT parasites to (*R*)-PA-824 in the intra-macrophage amastigote stage. WT (open circles) and RES III (closed circles) metacyclic promastigotes were used to infect starch-elicited, mouse peritoneal macrophages. Dose response curves are the non-linear regression fits using a four-parameter EC_50_ equation, yielding EC_50_ values of 2.1 ± 0.15 μM and > 50 μM for (*R*)-PA-824 against WT and RES III cells, respectively.
(TIF)

S2 Fig Levels of NTR2 in WT, RES III and NTR2 overexpressing promastigotes. WT (open circles) and RES III (closed circles) metacyclic promastigotes were used to infect starch-elicited, mouse peritoneal macrophages. Dose response curves are the non-linear regression fits using a four-parameter EC_50_ equation, yielding EC_50_ values of 2.1 ± 0.15 μM and > 50 μM for (*R*)-PA-824 against WT and RES III cells, respectively.
(TIF)

S1 Table List of proteins found to be consistently over- or under-expressed in RES III compared to WT promastigotes (see accompanying Excel document). (XLSX)

S2 Table List of SNPs and INDELs identified in drug resistant clones (see accompanying Excel document). (XLSX)

S3 Table Cloning primers used to generate over-expressing and knockout lines of *L*. *donovani* and recombinant expression in *E*.*coli*. (DOCX)
