# Supplementary material for: Activation of Bicyclic Nitro-drugs by a Novel Nitroreductase (NTR2) in Leishmania
Source: PLoS Pathog. 2016 Nov 3;12(11):e1005971. doi: 10.1371/journal.ppat.1005971 (PMC5094698; doi:10.1371/journal.ppat.1005971)
Supplement: S1 Text — (DOCX) [file ppat.1005971.s001.docx]

**Supporting Information**

**Procedures for the synthesis of DNDI-VL-2098 and CGI-17341.**

**General**

Chemicals and solvents were purchased from Sigma-Aldrich (UK), Alfa Aesar (UK), Apollo Scientific (UK), Fisher Chemicals (UK), TCI (UK) and VWR (UK). Air and moisture-sensitive reactions were performed under an inert atmosphere of nitrogen. Analytical thin-layer chromatography (TLC) used pre-coated TLC plates (layer 0.20 mm silica gel 60 with fluorescent indicator UV254, from Merck). Reaction products were visualised using a UV lamp (UV254/365 nm), and/or with chemical stains, where appropriate. Flash column chromatography was carried out using prepacked silica gel cartridges (230-400 mesh, 35-70 μm, from Teledyne ISCO) using a Teledyne ISCO CombiFlash Rf. ^1^H-NMR, ^13^C-NMR, ^19^F-NMR and 2D-NMR spectra were recorded on a Bruker Avance DPX 500 spectrometer (^1^H at 500.1 MHz, ^13^C at 125.8 MHz, ^19^F at 470.5 MHz), or a Bruker Avance III HD (^1^H at 400.1 MHz, ^13^C at 100.6 MHz). Chemical shifts (δ) are expressed in ppm recorded using the residual solvent as the internal reference in all cases. Signal splitting patterns are reported as singlet (s), doublet (d), triplet (t), quartet (q), multiplet (m), broad (br), or a combination thereof. Coupling constants (*J*) are quoted to the nearest 0.5 Hz. LC-MS analyses were performed with either an Agilent HPLC 1100 series connected to a Bruker Daltonics MicrOTOF or an Agilent Technologies 1200 series HPLC connected to an Agilent Technologies 6130 quadrupole LC/MS, where both instruments were connected to an Agilent diode array detector. LCMS chromatographic separations were conducted with either a Waters XBridge C18 column, 50 mm × 2.1 mm, 3.5 μm particle size, or Waters XSelect C18 column, 30 mm × 2.1 mm, 2.5 μm particle size; mobile phase, water/acetonitrile + 0.1% HCOOH, or water/acetonitrile + 0.1% NH_3_. High-resolution electrospray measurements were performed on a Bruker Daltonics MicrOTOF mass spectrometer. Preparative HPLC separations were achieved using a Waters mass-directed HPLC (system fluidics organizer, 2545 binary gradient module, 2×515 HPLC pumps, 2767 sample manager) connected in parallel to a Waters 3100 mass detector and a 2998 photodiode array detector. HPLC chromatographic separations were conducted using a Waters XBridge C18 column, 19 × 100 mm, 5 μm particle size; mobile phase, water/acetonitrile + 0.1% NH_3_.

**Synthesis of DNDI-VL-2098 ((*R*)-2-methyl-6-nitro-2-((4-(trifluoromethoxy)phenoxy)
methyl)-2,3-dihydroimidazo[2,1-*b*]oxazole).**

DNDI-VL-2098 was prepared in a single step from 4-(trifluoromethoxy)phenol and (*R*)-2-bromo-1-((2-methyloxiran-2-yl)methyl)-4-nitro-1*H*-imidazole (**II**) using a modification of the published synthesis of OPC-67683 (Sasaki *et al.*, 2006). Epoxide **II** itself was prepared from (2*R*)-2-methylglycidyl-4-nitrobenzoate (**I**) in 4 steps as previously described (Sasaki *et al.*, 2006) (Figure S2)

Solid NaH (60% suspension in oil, 12 mg, 0.3 mmol) was added to a solution of (*R*)-2-bromo-1-((2-methyloxiran-2-yl)methyl)-4-nitro-1*H*-imidazole (**II**) (66 mg, 0.25 mmol) and 4-(trifluoromethoxy)phenol (53 mg, 0.3 mmol) in anhydrous DMF (5 ml) at 0 °C. The reaction was allowed to warm to room temperature over 15 min and subsequently heated to 50°C for 19 h. The reaction was then added to satd. aq NaCl:EtOAc (1:1, 20 ml), the layers separated and the aqueous extracted with EtOAc (3×10 ml). The combined EtOAc layers were dried over MgSO_4_, filtered and the solvent removed under reduced pressure. The resultant crude product was purified by column chromatography (24 g silica, 20:80→100:0 EtOAc:heptane) to give the title compound as a white solid (32 mg, 32% yield). ^1^H-NMR (500 MHz, DMSO-*d*_6_) δ 8.15 (s, 1H, ArH), 7.31-7.27 (m, 2H, AA'BB', 2×ArH), 7.02-6.99 (m, 2H, AA'BB', 2×ArH), 4.38 (d, 1H, *J*=11.0 Hz, C*H*H), 4.34 (d, 1H, *J*=11.0 Hz, C*H*H), 4.31 (d, 1H, *J*=11.0 Hz, C*H*H), 4.19 (d, 1H, *J*=11.0 Hz, C*H*H), 1.69 (s, 3H, CH_3_). ^19^F-NMR (470 MHz, DMSO-*d*_6_) δ -57.4 (CF_3_). ^13^C-NMR (125 MHz, DMSO-*d*_6_) δ 157.3 (C), 156.0 (C), 146.1 (C), 142.8 (C), 123.0 (CH), 120.6 (q, *J*=254 Hz, CF_3_), 116.6 (CH), 116.4 (CH), 94.6 (C), 72.5 (CH_2_), 51.3 (CH_2_), 22.4 (CH_3_). MS (ES+): *m/z* 360 (100%) [M+H]^+^, 718 (45%) [2M+H]^+^, 741 (50%) [2M+Na]^+^. HRMS (ES+): calcd. for C_14_H_13_F_3_N_3_O_5_ [M+H]^+^ 360.0802, found 360.0801 (0.2 ppm).

**Synthetic strategy for synthesis of CGI-17341**

CGI-17341 was prepared by modification of a previous method (Nagarajan *et al.*, 1989). In this modification the explosive 2,4-dinitro-1*H*-imidazole was replaced with 2-bromo-4-nitro-1*H*-imidazole (**IV**). This alternative starting material proved to be considerably less reactive, and a two-step procedure was required to furnish CGI-17341 in low yield (Fig. S4).

**Synthesis of 1-(2-bromo-4-nitro-1*H*-imidazol-1-yl)butan-2-ol (intermediate IV).**

Neat 1,2-epoxybutane (901 mg, 12.5 mmol) was added to a suspension of 2-bromo-4-nitro-1*H*-imidazole (**III**) (960 mg, 5 mmol) and sodium acetate (410 mg, 5 mmol) in anhydrous EtOH (10 ml) and heated at 70 °C for 16 h. The reaction solvent was then removed under reduced pressure and the crude product purified directly by column chromatography (80 g silica, 0:100-100:0 EtOAc:heptane) to give the title compound as an off-white solid (1.01 g, 69% yield). ^1^H-NMR (500 MHz, CDCl_3_) δ 7.97 (s, 1H, ArH), 4.12 (d, 1H, *J*=11.5 Hz, C*H*H), 3.94-3.86 (m, 2H, C*H*H&CH), 3.41 (br s, 1H, OH), 1.63-1.50 (m, 2H, CH_2_), 1.04 (t, 3H, *J*=7.5 Hz, CH_3_). MS (ES+): *m/z* 264 (100%) [^79^Br M+H]^+^, 266 (93%) [^81^Br M+H]^+^.

**Synthesis of CGI-17341 ((*R*/*S*)-2-ethyl-6-nitro-2,3-dihydroimidazo[2,1-*b*]oxazole).**

Solid sodium hydride (60% in oil, 397 mg, 9.91 mmol) was added to a solution of alcohol I**V** (1.01 g, 3.81 mmol) in anhydrous dioxane at approximately 15 °C. The reaction was allowed to warm to room temperature, and subsequently heated to 102 °C with stirring for 6 h. The reaction mixture was then poured onto water (50 ml), and extracted with CH_2_Cl_2_ (3 × 50 ml). The combined CH_2_Cl_2_ layers were dried over MgSO_4_, filtered and the solvent removed under reduced pressure. The crude was then partially purified by flash column chromatography (120 g silica, 0:100→15:85 MeOH:CH_2_Cl_2_), before being purified by reverse phase HPLC (5:95→95:5 acetonitrile:water+0.1% NH_3_) to give the title compound as a tan solid (17 mg, 2.3% yield). ^1^H-NMR (400 MHz, CDCl_3_) δ 8.12 (s, 1H, ArH), 5.36-5.29 (m, 1H, CH), 4.41 (dd, 1H, *J*=11.0, 8.0 Hz, C*H*H), 3.98 (dd, 1H, *J*=11.0, 7.5 Hz, C*H*H), 1.90-1.83 (m, 2H, CH_2_), 0.96 (t, 1H, *J*=7.5 Hz, CH_3_). ^13^C-NMR (100 MHz, CDCl_3_) δ. 156.6 (C), 145.9 (C), 116.5 (CH), 89.6 (CH), 48.4 (CH_2_), 27.2 (CH_2_), 9.1 (CH_3_). MS (ES+): *m/z* 184 (100%) [M+H]^+^. HRMS (ES+): calcd. for C_14_H_14_F_3_N_2_O_3_ [M+H]^+^ 184.0717, found 184.0718 (-0.7 ppm).

**Synthesis of DNDI-VL-2098.** Synthesis of II from I in carried out in four steps (Sasaki *et al.*, 2006). Reagents and conditions: a) NaH, 4-OCF_3_-phenol, DMF, 50°C, 19 h, 32%.

**Synthesis of CGI-17341.**  Reagents and conditions: a) 1,2-epoxybutane, NaOAc, EtOH, 70°C, 16 h, 69%; b) NaH, dioxane, 102°C, 6 h, 2.3%.

**Synthesis of DNDI-VL-2098.** Synthesis of II from I in carried out in four steps (Sasaki *et al.*, 2006). Reagents and conditions: a) NaH, 4-OCF_3_-phenol, DMF, 50°C, 19 h, 32%.
