# Supplementary figures and images for: Activation of Bicyclic Nitro-drugs by a Novel Nitroreductase (NTR2) in Leishmania
Source: PLoS Pathog. 2016 Nov 3;12(11):e1005971. doi: 10.1371/journal.ppat.1005971 (PMC5094698; doi:10.1371/journal.ppat.1005971)

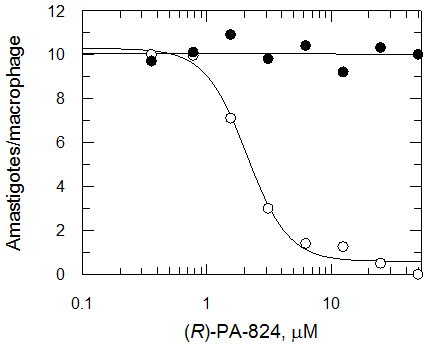

Supplement: S1 Fig — WT (open circles) and RES III (closed circles) metacyclic promastigotes were used to infect starch-elicited, mouse peritoneal macrophages. Dose response curves are the non-linear regression fits using a four-parameter EC50 equation, yielding EC50 values of 2.1 ± 0.15 μM and > 50 μM for (R)-PA-824 against WT and RES III cells, respectively. (TIF) [file ppat.1005971.s002.tif]

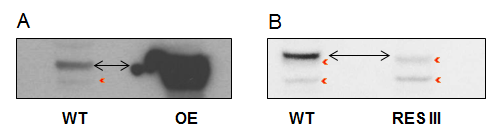

Supplement: S2 Fig — WT (open circles) and RES III (closed circles) metacyclic promastigotes were used to infect starch-elicited, mouse peritoneal macrophages. Dose response curves are the non-linear regression fits using a four-parameter EC50 equation, yielding EC50 values of 2.1 ± 0.15 μM and > 50 μM for (R)-PA-824 against WT and RES III cells, respectively. (TIF) [file ppat.1005971.s003.tif]
