# Supplementary material for: Activation of Bicyclic Nitro-drugs by a Novel Nitroreductase (NTR2) in Leishmania
Source: PLoS Pathog. 2016 Nov 3;12(11):e1005971. doi: 10.1371/journal.ppat.1005971 (PMC5094698; doi:10.1371/journal.ppat.1005971)
Supplement: S3 Table — (DOCX) [file ppat.1005971.s006.docx]

**S3 Table. Cloning primers used to generate over-expressing and knockout lines of *L. donovani* and recombinant expression in *E.coli***

Upper case letters refer to nucleotides corresponding to gene sequences in *L. donovani*; lower case refers to additional sequences used in generating constructs. Restriction endonuclease sites are underlined.

| **Construct** | **Primers** | **Sequence** |
| --- | --- | --- |
| **Ld*NTR2*^OE^** | Ld*NTR2*-BamHI_s | 5'-agatctATGTCCGCCGCTTCCAAGTCGATCGA-3' |
|  | Ld*NTR2*-BamHI_as | 5'-agatctCTACATGAATGGGTAGTCGTTGTAGC-3' |
|  | Ld*NTR2*- SmaI _s | 5'-cccgggATGTCCGCCGCTTCCAAGTCGATCGA-3' |
|  | Ld*NTR2*- XbaI _as | 5'-tctagaCATGAATGGGTAGTCGTTGTAGCCT-3' |
| **Ld*NTR2* DKO** | 5'UTR-NotI _s | 5´ataagaatgcggccgcTGTGCGCTGTTCTCGGGGACGTGA-3' |
|  | 5´UTR-HindIII/PmeI_as | 5´gtttaaacttacggaccgtcaagcttGGTGAGCGCTTTCCTCTTGCGAAT-3' |
|  | 3´UTR-PmeI/BamHI_s | 5´gacggtccgtaagtttaaacggatccCGCTCTCCTTGCCTGCCCGCGCC-3' |
|  | 3´UTR-NotI_as | 5´ataagtaagcggccgcCGATGCTGCCGAGATAGGACAACA-3' |
| **Ld*NTR2* recombinant** | Ld*NTR2*-NdeI_s | 5'-catATGTCCGCCGCTTCCAAGTCGATC-3' |
|  | Ld*NTR2*-BamHI_as | 5'-ggatccCTACATGAATGGGTAGTCGTTGTA-3' |
